# Comparison of inflammation, arterial stiffness and traditional cardiovascular risk factors between rheumatoid arthritis and inflammatory bowel disease

**DOI:** 10.1186/s12950-014-0029-0

**Published:** 2014-10-11

**Authors:** Fenling Fan, Abby Galvin, Lu Fang, David Andrew White, Xiao-lei Moore, Miles Sparrow, Flavia Cicuttini, Anthony Michael Dart

**Affiliations:** Department of Cardiovascular Medicine, The Alfred Hospital, Commercial Road, Melbourne, VIC 3004 Australia; Baker IDI Heart and Diabetes Institute, 75 Commercial Road, Melbourne, VIC 3004 Australia; Department of Gastroenterology, The Alfred Hospital, Commercial Road, Melbourne, VIC 3004 Australia; Department of Rheumatology, The Alfred Hospital, Commercial Road, Melbourne, VIC 3004 Australia; Department of Epidemiology and Preventative Medicine, Monash University, Melbourne, VIC 3800 Australia; Department of Cardiovascular Medicine, The 1st Affiliated Hospital of Medical College, Xi’an Jiaotong University, Xi’an, 710061 China

**Keywords:** Inflammation, Rheumatoid arthritis, Inflammatory bowel disease, Arterial stiffness, Pulse wave velocity

## Abstract

**Background:**

Inflammation plays an important role in the pathogenesis of atherosclerosis. The link between rheumatoid arthritis (RA) and an increased risk of cardiovascular disease and mortality is well established; however, the association between inflammatory bowel disease (IBD) and cardiovascular risk is controversial. Arterial stiffness is both a marker and risk factor for atherosclerosis. Here we aimed to 1) compare circulating markers of inflammation and endothelial dysfunction, traditional cardiovascular risk factors, and arterial stiffness between RA and IBD to help to understand their different associations with cardiovascular disease; 2) assess the impacts of circulating markers of inflammation and endothelial dysfunction, and traditional risk factors on arterial stiffness.

**Methods:**

Patients with RA (n = 43) and IBD (n = 42), and control subjects (n = 73) were recruited. Plasma inflammatory markers and von Willebrand factor (vWF) were measured by Multiplex assays or ELISA. Arterial stiffness was determined by brachial-ankle pulse wave velocity (baPWV) and ankle-brachial index (ABI) was measured. Framingham Risk Score (FRS) was calculated, and other traditional risk factors were also documented.

**Results:**

Plasma levels of several inflammatory markers and vWF were significantly but comparably elevated in RA and IBD compared with controls, except for a higher level of C-reactive protein (CRP) in RA than IBD. Compared to controls, FRS, body mass index, waist circumference, and triglycerides were increased in RA, but not in IBD. baPWV did not significantly differ among 3 groups, while ABI was modestly but significantly lower in IBD than controls. Circulating markers (macrophage migration inhibitory factor, tumour necrosis factor-α, CRP, and vWF) were significantly associated with baPWV. However, traditional risk factors (age, systolic blood pressure, body mass index, diabetes and triglycerides) were the parameters associated with baPWV in multiple regression analyses (overall r = 0.866, p < 0.001).

**Conclusions:**

RA has a higher level of CRP and more pronounced traditional cardiovascular risk factors than IBD, which may contribute to the difference in their associations with cardiovascular disease and mortality. Traditional risk factors, rather than inflammation markers, are major predictors of arterial stiffness even in subjects with inflammatory disorders. Our results point to the importance of modifying traditional risk factors in patients with inflammatory disorders.

## Introduction

It is well known that inflammation plays a pivotal role in the pathogenesis and progression of atherosclerosis and that measures of systemic inflammation, such as circulating C-reactive protein (CRP) and interleukin (IL)-6, provide useful prognostic information for atherosclerosis [1-4]. Chronic inflammatory diseases such as rheumatoid arthritis (RA) have been strongly linked to accelerated atherosclerosis and increased incidence of cardiovascular events and mortality [5-8]. Such increased cardiovascular risk in RA cannot be completely explained by traditional cardiovascular risk factors [5]. In patients with inflammatory bowel disease (IBD, ulcerative colitis (UC) and Crohn’s disease (CD)), however, association with the risk of cardiovascular disease is controversial. While the majority of studies showed no evidence for an increased risk of cardiovascular disease and mortality in IBD [9-11], this is not a universal finding [12]. In chronic inflammatory conditions, proinflammatory cytokines and a systemic inflammatory state play a crucial role in accelerated atherosclerosis and the development of cardiovascular events. The chronic and systemic inflammation can induce endothelial activation and dysfunction, which may amplify atherosclerosis in chronic inflammatory conditions. Whether there are differences in proinflammatory cytokines and endothelial dysfunction markers which can account for any differences in their susceptibility to atherosclerosis is yet to be explored.

Large artery stiffness is known to be increased in patients with atherosclerosis and it is both a surrogate marker and an independent risk factor for atherosclerosis [13-15]. Aortic PWV (commonly the carotid-femoral PWV) is accepted as the “gold standard” measure of arterial stiffness. Brachial-ankle PWV (baPWV) is, however, a promising technique to measure arterial stiffness conveniently and more suited to routine clinical use than some other more intrusive or complicated approaches [16,17]. In addition, the presence of vascular morphological changes, such as increased carotid intima-media thickness and the presence of carotid plaque, and the consequences of stenosis, such as reduced ankle-brachial index (ABI), have also been found to have diagnostic and predictive value [18,19]. In chronic inflammatory diseases, elevated proinflammatory cytokines may promote vascular changes and increase arterial stiffness, and subsequently contribute to increased risk for cardiovascular disease. Whether these two diseases elicit different vascular changes is unclear.

We therefore aimed to compare circulating markers of inflammation and endothelial dysfunction, and arterial properties (baPWV, upstroke time (UT) of the ankle pulse pressure and ABI), as well as traditional risk factors between RA and IBD in an attempt to understand the different associations with cardiovascular disease between these two inflammatory conditions. Furthermore, we aimed to examine the impacts of traditional risk factors, and circulating markers of inflammation and endothelial dysfunction on vascular changes to better understand the underlying mechanism for accelerated atherosclerosis in inflammatory conditions.

## Materials and methods

The study was approved by the Human Ethics Research Committee of the Alfred Hospital and all subjects provided written informed consent prior to their participation. All clinical investigation was conducted according to the principles expressed in the Declaration of Helsinki.

### Subjects

Patients with RA (n = 43) and IBD (n = 42, UC: 40% and CD: 60%) were recruited from the respective clinics at the Alfred Hospital. RA patients fulfilled the diagnostic criteria for the presence of RA proposed by the American Rheumatism Associations [20]. The diagnosis of IBD was established by gastroenterologists based on clinical, laboratory, radiological, endoscopic and histological criteria. Patients in our study were assessed at disease specific specialist clinics of the Alfred Hospital by Royal Australian College of Physicians accredited specialists. Age- and gender-matched healthy volunteers (n = 73) were recruited from the risk evaluation clinic of Baker IDI Heart and Diabetes Institute. Patients and controls were older than 35 years. Subjects with known cerebrovascular, coronary or peripheral vascular disease were excluded. Subjects’ demographics, medical and medication history were documented. Among RA patients, disease activity was assessed by the severity of joint pain, stiffness, and swelling. Disease activity in CD patients was assessed by the Harvey Bradshaw Index (HBI), while in UC patients was assessed by the criteria of “Truelove-Witts”. The majority of patients with RA or IBD received disease modifying therapy in addition to symptomatic treatment. Common treatments for the IBD group at the time of their vascular study included azathiaprine (n = 11, 26%), sulphasalazine (n = 9, 24%), melasamine (n = 9, 24%), 6-mercapto-purine (n = 6, 14%) and biologics (n = 6, 14%). Amongst the RA group common treatments included methotrexate (n = 17, 40%), biologics (n = 12, 28%), and plaquenil (n = 5, 12%). Biologics include tumour necrosis factor-α (TNF-α) and IL-6 antagonists. Other treatments included systemic and/or local corticosteroids (RA: n = 11, 25.6%, and IBD: n = 7, 16.7%), and non-steroidal anti-inflammatory agents (RA: n = 3, 7% and IBD: 0%).

### Vascular measures

baPWV, UT of pulse wave and ABI were measured in a quiet environment using an automated oscillometric machine, the Colin VP-1000 Plus. A phonocardiogram sensor was placed over the mid-point of patients’ third ribs and electrocardiogram sensors on the anterior aspect of each wrist. baPWV was measured simultaneously and bilaterally with the use of these sensors and four blood pressure cuffs with multihead pressure transducers placed over the brachial and posterior tibial arteries. Based on patients’ height, baPWV was calculated using the assessment of pulse transit time between the brachial and posterior tibial arteries. Similarly, ABI was calculated simultaneously and bilaterally as the ratio of the systolic blood pressure of the posterior tibial and brachial arteries. Two measurements of ABI and baPWV were taken, and the average of the two recorded. Analyses of baPWV and UT were undertaken using the average of the left and right whilst for ABI analyses were performed using the lowest value.

### Traditional risk factors

Fasting plasma lipids (total cholesterol, triglycerides, low-density lipoprotein cholesterol (LDL-C) and high-density lipoprotein cholesterol (HDL-C)) and glucose were determined at the Department of Chemical Pathology of the Alfred Hospital. The blood pressures reported were all obtained in the morning after an overnight fast and at the same visit as their vascular assessment. Blood pressures were measured in the seated position using an automated sphygmomanometer (HEM-907; Omron). Three readings, one minute apart, were taken and the average of last 2 readings was used. If the first reading was abnormal, then the second reading was 4 minutes apart. For each participant, a FRS for 10-year ‘hard’ coronary heart disease endpoints was calculated (see http://cvdrisk.nhlbi.nih.gov/calculator.asp).

### Plasma markers

Plasma levels of TNF-α, IL-6, IL-1β, IL-10, and von Willebrand factor (vWF) were measured using multiplex kits from Millipore according to the manufacturer’s instruction. The appropriate cytokine standards, plasma samples (25 μL), and fluorescent conjugated, antibody-immobilized beads were added to wells of a pre-wet filtered plate and then incubated overnight at 4°C. The following day, the plate was washed twice with wash buffer and then incubated with secondary detection antibody for 1 h, followed by subsequent incubation with strepavidin-PE for 30 min. After the plate was washed twice again with wash buffer, it was run on the Luminex system (Biorad) with the addition of sheath fluid. Concentrations of different analytes in the plasma samples were determined by using respective standard curves generated in the multiplex assays. Neat plasma samples were used for all assays except for vWF (1:10,000 dilutions, using assay buffer provided in the kit).

Macrophage migration inhibitory factor (MIF) in plasma was measured using commercial ELISA kits (R&D System) according to the manufacturer’s instructions. All samples and standards were measured in duplicate and averages used. High-sensitivity CRP in plasma was measured at the Department of Chemical Pathology of the Alfred Hospital.

E-selectin^+^ endothelial microparticles (EMPs) were measured by flow cytometry in plasma. In brief, plasma was thawed completely and centrifuged at 16,000 g for 5 min at 4°C to deplete platelets or any cell debris. 100 μL aliquot was incubated with 10 μL of FITC-labelled anti-human E-selectin antibody (clone: BBIG-E5, R & D Systems) or a corresponding mouse IgG isotype (R & D Systems) at room temperature for 20 min with gentle shaking. At the end of incubation, 300 μL of double filtered 0.2% FBS/PBS (filtered through a 0.2 μm and then a 0.1 μm membrane filter) was added and samples were counted with a Canto II flow cytometer (BD Biosciences) for 5 min. Megamix beads (Biocytex, France), a mixture of 0.5 μm, 0.9 μm, and 3 μm beads, were used for size calibration according to the manufacture’s instruction. For EMP enumeration, 30 μL of diluted calibration beads (BD Biosciences, USA) was added to each FACS tube and a formula was used based on the concentration of the added calibration beads [21], which discriminated themselves from the EMP population on the FSC-SSC cytogram. All counting data were then analysed using FlowJo software (Tree Star). Results were presented as counts of E-selectin^+^ EMP per μL of plasma.

### Statistical analysis

Continuously variable data are presented as mean ± SD for normally distributed data or median (25, 75 percentile) for non-normally distributed data. Categorical data is presented as percentage, and analysed by χ2. Continuously variable data were evaluated for normality using the Kolmogorov-Smirnov (K-S) test. Between-group analysis was performed by ANOVA, followed by scheffé test for normally distributed data, or by Kruskal-Walis test for non-normally distributed data. Bivariate correlations were assessed with Pearson correlation coefficient for normally distributed data and Spearman rank correlation coefficient for non-normally distributed data, respectively. Multiple regression analysis was undertaken using stepped entry and removal with Pin set at p = 0.1 and Pout at p = 0.05. Skewed data was Ln transformed prior to inclusion in multiple regression analyses. All statistical analyse were performed using SPSS v19 and statistical significance was set at p < 0.05 (two-sided).

## Results

### Demographic characteristics and traditional risk factors

The groups were well matched by age and gender. Systolic and diastolic blood pressure, body mass index (BMI), waist circumference and triglycerides were significantly higher in patients with RA compared to controls. Smoking was more prevalent in both patient groups compared with controls. Diastolic blood pressure was significantly higher in IBD patients compared with controls. More RA patients received anti-hypertensive treatment than controls or IBD patients. FRS was significantly elevated in RA group but not in IBD compared with the control group (Table 1). Mean disease duration of RA patients was 9.2 ± 7.9 years. The majority of RA patients were either asymptomatic or experiencing only mild symptoms at the time of their assessment. Only 17.5% of RA patients were experiencing moderate or severe symptoms. Mean disease duration of IBD patients was 11.5 ± 10.7 years. In CD group, average disease score was 2.4, while in UC group, all but one patient had mild disease at the time of their cardiovascular assessment. Thus, at the time of assessment, most patients were effectively treated with regard to disease signs and symptoms.

**Table 1:** **Demographic characteristics of subjects**

	**Control (n = 73)**	**RA (n = 43)**	**IBD (n = 42)**
Age, y	51 ± 10	55 ± 11	50 ± 10
Male, %	46	51	45
SBP, mmHg	118 ± 14	126 ± 13*	125 ± 17
DBP, mmHg	67 ± 10	74 ± 9**	74 ± 10**
Body mass index, kg/m^2^	25.6 ± 3.8	28.1 ± 5.8*	26.9 ± 6.1
Waist, cm	88.2 ± 11.7	96.0 ± 15.5*	92.3 ± 14.3
Diabetes, %	10	14	10
Total cholesterol, mmol/L	5.45 ± 0.86	5.30 ± 1.13	5.23 ± 1.20
LDL-C, mmol/L	3.45 ± 0.81	3.25 ± 1.12	3.19 ± 1.15
HDL-C, mmol/L	1.50 ± 0.33	1.44 ± 0.37	1.46 ± 0.45
Triglycerides, mmol/L	0.9 (0.7, 1.4)	1.30 (0.9, 1.6)**	1.0 (0.7, 1.7)
Never/ex/current smokers, %	77/19/4	40/34/26***	28/52/20***
Anti-HT treatment, %	8	37***	9##
LLT, %	4	11	5
FBS, mmol/L	4.8 (4.5, 5.2)	4.9 (4.5, 5.4)	4.6 (4.4, 5.2)
FRS	2.0 (0, 4.5)	4.0 (1.0, 12.0)**	2.0 (0, 8.0)

Results were expressed as mean ± SD, or percentage or median (25, 75 percentile). *,**,*** vs. control p < 0.05, <0.01, <0.001, respectively, ##, vs. RA p < 0.01. Anti-HT treatment: anti-hypertensive treatment; ex-smoker: prior smokers who have not smoked in the last 12 months; DBP: diastolic blood pressure; FBS: fasting blood sugar; FRS: Framingham Risk Score; HDL-C: high-density lipoprotein cholesterol; IBD: inflammatory bowel disease; LDL-C: low-density lipoprotein cholesterol; LLT: lipid-lowering therapy; RA: rheumatoid arthritis; SBP: systolic blood pressure.

### Inflammatory and endothelial markers, and vascular properties

Levels of CRP, IL-1β, IL-6, IL-10, TNF-α, MIF and vWF were elevated in both RA and IBD patients compared with controls (Table 2). Whilst there was a tendency for E-selectin^+^EMP to be elevated in the RA group, the differences failed to achieve statistical significance. The elevation of most inflammatory markers and vWF did not differ significantly between RA and IBD, but the level of CRP was significantly higher in RA compared to IBD. Since data were non-normally distributed, we also did comparisons on Ln transformed data. The results of statistical significance were almost identical to those presented in Table 2. The only exception was that the difference in Ln transformed E-selectin + EMP between RA and IBD reached significant (p = 0.036).

**Table 2:** **Change of circulating inflammatory and endothelial markers**

	**Control**	**RA**	**IBD**
CRP, μg/mL	0.90 (9.50, 2.00)	3.8 (0.7, 7.9)***	1.70 (0.70, 4.05)*#
IL-1β, pg/mL	0.74 (0.31, 1.23)	0.87 (0.36, 2.95)*	0.71 (0.32, 1.81)
IL-6, pg/mL	0.52 (0.33, 0.80)	1.52 (0.54, 3.37)***	1.03 (0.56, 1.79)***
IL-10, pg/mL	11.4 (6.6, 18.6)	15.8 (10.7, 38.7)*	19.3 (12.5, 31.3)***
TNF-α, pg/mL	2.32 (1.46, 3.75)	3.29 (1.99, 6.10)*	3.27 (2.18, 4.22)**
MIF, ng/mL	25.7 (21.4, 33.1)	49.3 (39.5, 79.0)**	46.2 (37.6, 58.2)**
vWF, μg/mL	7.56 (5.25, 12.50)	10.83 (7.68,19.36)**	11.32 (7.75,18.15)**
E-selectin + EMP, (/μL)	154 (122, 185)	164 (129, 204)	132(109, 184)

Results were expressed as median (25, 75 percentile). *, **, *** vs. control (p < 0.05, <0.01, <0.001), # vs. RA (p < 0.05). CRP: C-reactive protein; EMP: endothelial microparticles; IBD: inflammatory bowel disease; IL-1β: interleukin-1β; IL-6: interleukin-6; IL-10: interleukin-10; MIF: macrophage migration inhibitory factor; RA: rheumatoid arthritis; TNF-α: tumour necrosis factor-α; vWF: von Willebrand factor.

MIF was increased in former or current smokers compared with non-smokers (48.6 ng/mL (36.2, 59.9 ng/mL) and 41.8 ng/mL (30.2, 59.1 ng/mL) vs. 33.3 ng/mL (25.4, 48.7 ng/mL), p = 0.026). Vascular measures for the 3 groups are shown in Table 3. The minimum value of ABI was modestly but significantly lower in IBD than controls but other changes were not significant.

**Table 3:** **Comparisons of baPWV, ABI, and UT**

	**Control**	**RA**	**IBD**
baPWV (m/s)	1345 ± 264	1437 ± 216	1382 ± 259
ABI	1.11 ± 0.06	1.10 ± 0.08	1.07 ± 0.07*
UT (ms)	139.8 ± 14.0	143.1 ± 14.0	140.2 ± 17.6

Results were expressed as mean ± SD.*vs. control (p < 0.05). ABI: ankle-brachial index; baPWV: brachial-ankle pulse wave velocity; IBD: inflammatory bowel disease; RA: rheumatoid arthritis; UT: upstroke time of pulse wave.

### Correlations between inflammatory markers, vascular properties, and traditional risk factors

**Figure 1:**
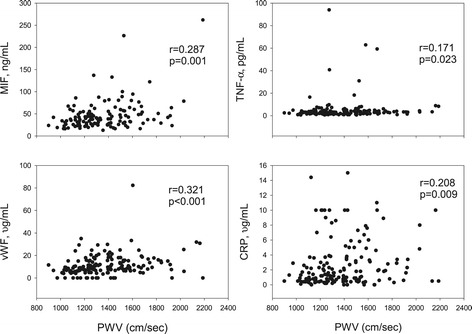
**The correlations between circulating markers and brachial-ankle pulse wave velocity (PWV).** Correlation was assessed by Spearman rank Correlation. CRP: C-reactive protein; MIF: macrophage migration inhibitory factor; TNF-α: tumour necrosis factor-α; vWF: von Willebrand factor.

**Figure 2:**
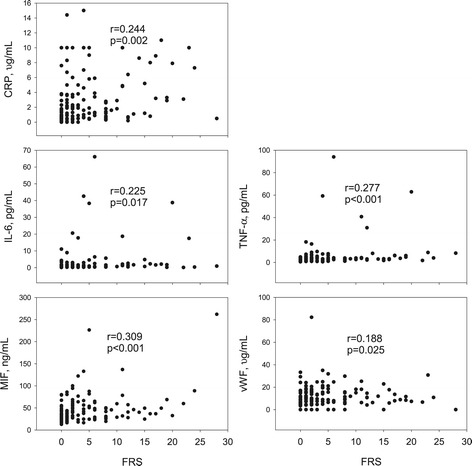
**The correlations between circulating markers and the 10 year Framingham risk score (FRS).** Correlation was assessed by Spearman rank Correlation. CRP: C-reactive protein; IL-6: interleukin-6; MIF: macrophage migration inhibitory factor; TNF-α: tumour necrosis factor-α; vWF: von Willebrand factor.

In the whole population, significant correlations were found between baPWV and MIF, TNF-α, CRP, and vWF (Figure 1). Neither UT nor ABI correlated with any of plasma markers. FRS also significantly correlated with CRP, IL-6, TNF-α, MIF, and vWF (Figure 2). Significant correlations existed between several inflammatory markers (and vWF) and classical risk factors (Table 4). The inflammatory markers were also highly auto-correlated (data not shown).

**Table 4:** **Correlations between inflammatory and endothelial markers and traditional risk factors**

	**CRP**	**IL-1β**	**IL-6**	**IL-10**	**TNF-α**	**MIF**	**E-sel + EMP**	**vWF**
Age	0.081	-0.013	0.157	0.160*	0.005	0.136	0.018	0.240**
SBP	0.324***	0.110	0.203*	0.235*	0.163*	0.303**	0.094	0.154
BMI	0.403***	-0.017	0.047	-0.029	0.049	0.072	0.186*	0.136
TG	0.254**	-0.034	0.034	-0.043	-0.085	-0.263**	0.147	0.180*

*,**,*** indicate significance at p < 0.05, <0.01, <0.001, respectively. BMI: body mass index; CRP: C-reactive protein; EMP: endothelial microparticles; IBD: inflammatory bowel disease; IL-1β: interleukin-1β; IL-6: interleukin-6; IL-10: interleukin-10; MIF: macrophage migration inhibitory factor; RA: rheumatoid arthritis; SBP: systolic blood pressure; TG: triglycerides; TNF-α: tumour necrosis factor-α; vWF: von Willebrand factor. Statistically significant correlations are shown with bold typeface.

### Prediction of baPWV, ABI, and UT

baPWV correlated with age (r = 0.737, p < 0.001), systolic blood pressure (r = 0.616, p < 0.001) and triglycerides (r = 0.311, p < 0.001). baPWV was higher in diabetic subjects than non-diabetic subjects (1570 ± 298 m/s vs. 1360 ± 248 m/s, p = 0.001). In multiple regression analyses, significant determinants of baPWV were age, systolic blood pressure, BMI, diabetes and triglycerides with overall r value of 0.866 (p < 0.001). Stepwise addition of each inflammatory or endothelial marker to regression analyses including the above 5 traditional risk factors was undertaken, but none were independently related to baPWV. Disease group (either RA or IBD) was not a significant determinant of baPWV.

In addition, patients on anti-hypertensive treatment (n = 26) had higher average baPWV than those not on anti-hypertensive treatment (n = 132) (1535 ± 48 vs. 1352 ± 22 (m/s), p = 0.001). Those on anti-hypertensive treatment were older (59.6 ± 2.1 vs. 51.1 ± 0.9 years, p = 0.000), having higher SBP (132 ± 2.7 vs. 120.9 ± 1.3 mmHg, p = 0.01), and greater BMI (29.5 ± 1.1 vs. 26.0 ± 0.4, p = 0.002). They were also more likely diabetic (26.9% vs. 7.5%, p = 0.009), and tended to have higher TG (1.44 ± 0.11 vs. 1.15 ± 0.06, p = 0.06). Because of the associations shown above, anti-hypertensive treatment was not significantly related to PWV after adjusting for age, SBP, BMI, DM and TG in the multiple regression analysis.

ABI was lower in women than men (1.08 ± 0.06 vs. 1.11 ± 0.07, p = 0.003) and in current smokers compared with ex- and non-smokers (1.06 ± 0.07 vs. 1.10 ± 0.07). UT was higher in women than men (145.2 ± 16.8 ms vs. 134.7 ± 10.2 ms, p <0.001) and in current and former smokers vs. non-smokers (144.8 ± 15.1 ms and 144.1 ± 16.8 ms vs. 137.9 ± 13.3 ms, p = 0.029). In regression analyses in the whole cohort, ABI was dependent on gender and smoking (with an overall r = 0.312, p < 0.001) as was UT (r = 0.425, p < 0.001) but neither was correlated to any of the inflammatory or endothelial markers. Disease group (either RA or IBD) was a significant determinant for ABI but not UT.

## Discussion

In the present study, we have made several main observations. First, plasma levels of several inflammatory markers and vWF were significantly but comparably elevated in RA and IBD compared with controls, except for a higher level of CRP in RA than IBD. Next, traditional cardiovascular risk factors were more pronounced in RA than IBD. Furthermore, whilst ABI was modestly but significantly lower in IBD compared to controls, there were no significant differences in baPWV amongst the 3 groups. baPWV was mainly associated with traditional risk factors, with age and systolic blood pressure making the most significant contribution to baPWV. CRP, MIF, TNF-α and vWF significantly correlated with baPWV, but the correlations disappeared after adjusting for main traditional risk factors. We also observed significant associations between traditional risk factors and several inflammatory markers.

It is well established that patients with RA are at an increased risk of developing cardiovascular events and mortality [6-8], however, the association between cardiovascular disease and IBD is controversial. While some studies showed that IBD was not related to an increased risk of cardiovascular disease and mortality [9-11,22], other more recent studies supported such association [12,23-25]. A recent meta-analysis that included 33 studies enrolling 207, 814 patients and 5,774,898 controls has concluded that patients with IBD are at major risk for venous thromboembolism and mesenteric ischemia and, to a lesser degree, arterial thromboembolism and ischemic heart disease. However, IBD is not associated with an increase in the risk of cardiovascular mortality [26]. In the present study, we compared markers of inflammation and endothelial dysfunction, traditional cardiovascular risk factors, and baPWV and ABI between RA and IBD. The elevation of a number of inflammation markers and vWF did not differ significantly between RA and IBD, but the level of CRP was significantly higher in RA than IBD. Notably, CRP level is known to be affected by anti-inflammation treatment such as biologics. However, CRP still remained higher in RA than IBD even though more RA patients received biologics than IBD patients at the time of their vascular study. It is well known that CRP is both a predictor and an independent risk factor for cardiovascular disease and is a predictor of cardiovascular mortality in patients with RA [27]. CRP < 1.0 μg/mL is generally regarded as low risk, 1.0-2.99 μg/mL as intermediate risk and >3 μg/mL as high risk for the development of cardiovascular disease in subjects apparently free of such disease at baseline. In the current cohort, 55.8% of RA patients and 26.2% of IBD patients had CRP > 3 μg/m in comparison with only 12.3% in the control group. So, while our results confirm chronic systemic inflammation in both RA and IBD, we do observe a higher plasma level of CRP in RA, which may have some pathological impact on cardiovascular disease. In addition, although not statistically significant, IL-6 was 47.6% higher in RA than in IBD. IL-6 levels also show a graded relationship to future cardiac risk [28] with subjects in the 3^rd^ and 4^th^ quartile having relative risks of future events of 2.2-3.5 times and cutoff values being 1.47 and 2.28 pg/mL, respectively. In the current cohort, 53% and 36.4% of RA patients had IL-6 > 1.47 pg/mL and >2.28 pg/mL, respectively, whereas 32.4% and 18.4% of IBD patients had IL-6 > 1.47 pg/mL and IL6 > 2.28 pg/mL, respectively.

We also found that traditional cardiovascular risk factors were more prevalent in RA compared to IBD. FRS, calculated based on age, male gender, smoking, systolic blood pressure, total cholesterol, HDL-C, and anti-hypertensive treatment, was significantly increased in RA but not in IBD, compared with the control group. BMI, waist size, and triglycerides were also increased only in RA, compared to controls. Previous studies have also documented increased prevalence of traditional risk factors in RA [6,29]. In contrast, only two traditional risk factors i.e. hypertension and smoking existed in IBD patients. Consistent with our study, FRS was not increased in IBD in a previous study [30]. Other than hypertension, elevated traditional risk factors have not been confirmed in IBD [31]. BMI and LDL-C were even lower in IBD [32], which may be due to intestinal malabsorption. So, while chronic systemic inflammation in IBD supports its association with cardiovascular risk, some unique features related to traditional risk factors in IBD may alter such association. Take together, more pronounced traditional risk factors and a higher level of CRP in RA, compared to IBD, may contribute to the different associations with ischemic heart disease and cardiovascular mortality between RA and IBD, reported in literature. Meanwhile, different focuses should be placed when monitoring and modifying cardiovascular risk factors in patients with RA and IBD.

We also observed significant associations between inflammation and traditional risk factors. Several inflammatory markers significantly correlated with FRS, as well as with individual traditional risk factor. Notably, the associations with CRP were strongest. Given the cross-sectional nature of this study, it is impossible to determine whether these associations are causal. However, inflammation has been suggested to play a role in the pathogenesis of hypertension [33]. There is evidence showing that inflammation causes obesity [34]. Insulin resistance is known to be induced by inflammation arising from adipose tissue [35,36] and elevated levels of CRP and IL-6 are shown to predict the development of diabetes [37]. Inflammation also induces decreased HDL-C and subsequent hypertriglyceridemia [38]. Reverse cholesterol transport, a protective process against atherosclerosis, is adversely affected by inflammation [39]. So, the elevations in inflammatory markers may partly underlie the elevation seen in traditional risk factors. The elevation of CRP in RA could contribute to elevated traditional risk factors and increased susceptibility to cardiovascular disease in RA.

Arterial stiffness is both a marker and a risk factor for cardiovascular disease [13-15] and inflammation plays an important role in the pathogenesis of arterial stiffness. Increased aortic PWV was reported in patients with RA in a number of studies [40,41] and in patients with IBD in a small study [42]. However, we did not observe a significant increase in mean baPWV in either RA or IBD. Similarly, some other studies did not find increased PWV in RA [43], or in IBD [32]. Our results may suggest that increased traditional risk factors, but not the effect of systemic inflammation on arterial stiffness, are more likely to underlie the reported increased risk of cardiovascular disease in RA. Previous studies have shown associations between CRP and PWV in healthy subjects [44], hypertensive [45] and RA patients [40]. The increase in aortic PWV in RA was reduced by anti-TNF-α or anti-IL-6 therapy without change in blood pressure [40,41]. In the present study, we found that CRP, MIF, TNF-α and vWF significantly correlated with baPWV, however, the correlations disappeared after adjusting for main traditional risk factors. The major univariate determinants of baPWV were age (r = 0.737) and systolic blood pressure (r = 0.616), and after including BMI, triglycerides and diabetes, the overall r was 0.866, suggesting that traditional cardiovascular risk factors, rather than inflammatory markers or vWF, are predictors of baPWV. However, in healthy subjects without inflammatory disease, associations between CRP and PWV (including baPWV) existed after adjustment for traditional risk factors [44,46]. The clear associations between traditional risk factors and inflammatory markers in this study likely account for, in part, the failure of the inflammatory markers to be statistically significant in multiple regression. Moreover, medication, particularly disease modifying drugs, which may reduce inflammation and stiffness, probably weaken the link between inflammation and arterial stiffness. Taken together, in chronic inflammatory patients without established cardiovascular disease, arterial stiffness is associated with traditional risk factors, pointing to the importance of modifying traditional risk factors in inflammatory disorders.

The prognostic significance of peripheral arterial disease is well recognised. The accepted ABI demarcation of abnormality is ≤0.9 and prognosis for vascular events and total mortality becomes progressively worse with lower ABI values. However values of 0.9-1.1 are also predictive of increased mortality [47] and likely represent the evidence of early vascular disease. We found that ABI was modestly but significantly lower in IBD patients than controls, which may suggest a higher incidence of early stage of peripheral arterial disease in IBD than RA. Significant determinants of ABI were limited to gender and smoking but not any of inflammatory markers. Peripheral arterial disease is known to be particularly related to cigarette smoking [48] and it is noteworthy that the IBD group had the lowest percentage of subjects who had never smoked.

This study has several limitations. First, we measured baPWV to assess arterial stiffness. We cannot exclude the possibility that the results from aortic PWV may be different. However, baPWV has been shown to strongly correlate with carotid-femoral PWV with r values of 0.73-0.79 being reported [49-51]. In multiple regression analysis about 58% of the variance of baPWV was attributable to carotid-femoral PWV and about 23% to femoral-ankle PWV [51]. Age and blood pressure are major and similar determinants of both carotid-femoral PWV and baPWV. Whilst carotid-femoral PWV is determined by the properties of the aorta, baPWV also includes the peripheral arterial circulation which may contribute to its ability to act as a risk marker. Thus, correlation with FRS was 0.48 for carotid-femoral PWV and 0.63 for baPWV although the two measures exhibited comparable predictive values for stroke and coronary artery disease [49]. A meta-analysis of more than 8000 subjects confirmed the predictive value of baPWV for cardiovascular disease. An increase in baPWV of 1 m/s corresponded to an increase of 12%, 13% and 6% in total cardiovascular events, cardiovascular mortality and total mortality, respectively [52]. Thus, whilst use of baPWV could be a potential limitation of the study there is substantial evidence that baPWV does reflect the response of the arteries to risk factors. In addition, baPWV is a simple and automatic measure, which can be done in an outpatient clinic setting, as part of routine care. Another potential limitation of the study is relatively small group size especially for the disease groups. Despite this, significant differences in traditional risk factors and CRP were evident between the groups and the overall r value for baPWV in multiple regression analysis was > 0.86.

In conclusion, RA has a higher level of CRP and more pronounced traditional risk factors, compared to IBD, which may contribute to the different associations with cardiovascular disease and mortality reported. Traditional risk factors, but not inflammatory markers, are the major parameters associated with arterial stiffness even in a cohort with evident systemic inflammation. Our data suggest that it is important to control traditional risk factors in patients with these inflammatory disorders.

## Abbreviations

ABI: Ankle-brachial index
baPWV: Brachial-ankle pulse wave velocity
BMI: Body mass index
CD: Crohn’s disease
CRP: C-reactive protein
IBD: Inflammatory bowel disease
RA: Rheumatoid arthritis
UC: Ulcerative colitis
UT: Upstroke time of pulse wave
